# Left ventricular remodeling index to predict ventricular tachyarrhythmia in dilated cardiomyopathy with ejection fraction < 35%

**DOI:** 10.1186/s13244-025-02059-6

**Published:** 2025-08-29

**Authors:** Xi Jia, Weipeng Yan, Xuan Ma, Zhixiang Dong, Jiaxin Wang, Shujuan Yang, Kankan Zhao, Zhuxin Wei, Yun Tang, Pengyu Zhou, Xingrui Chen, Yujie Liu, Xiuyu Chen, Shihua Zhao

**Affiliations:** 1 Department of Magnetic Resonance Imaging, Fuwai Hospital, National Center for Cardiovascular Diseases of China, Chinese Academy of Medical Sciences and Peking Union Medical College, Beijing, China; 2 School of Biomedical Engineering and Informatics, Nanjing Medical University, Nanjing, China

**Keywords:** Dilated cardiomyopathy, Cardiac magnetic resonance, Remodeling index, Ventricular tachyarrhythmia

## Abstract

**Objectives:**

To assess the left ventricular remodeling index (LVRI) for predicting ventricular tachyarrhythmia (VTA) in patients with dilated cardiomyopathy (DCM) with left ventricular ejection fraction (LVEF) < 35%.

**Materials and methods:**

In this retrospective single-center study, consecutive DCM patients with LVEF < 35% (*n* = 271) who underwent cardiac magnetic resonance (CMR) imaging were followed up. The study endpoint was VTA, including sudden cardiac death and major ventricular arrhythmias. The CMR-derived LVRI was defined as the cubic root of the LV end-diastolic volume divided by the maximal LV wall thickness. Competing risk regression analysis and Kaplan–Meier analysis were used to evaluate the association of LVRI with VTA.

**Results:**

Over 71-month median follow-up (interquartile range: 17–134 months), 35 (12.9%, mean age 46.7 years, 27 males) participants reached VTA events. The presence (62.9% vs. 60.2%, *p* = 0.761) and extent (6.9 ± 6.6 vs. 6.5 ± 8.3, *p* = 0.747) of late gadolinium enhancement (LGE) and LVEF (23.3 ± 6 vs. 21.9 ± 10.3, *p* = 0.197) were not significantly different between the patients with and without endpoint. Kaplan–Meier curve analysis showed that participants with LVRI ≥ 7.5 were more likely to experience VTA (*p* < 0.0001). In the multiple competing risk analysis, LVRI ≥ 7.5 (HR, 2.496; 95% CI: 1.213–5.138; *p* = 0.013) was observed as an independent predictor of VTA after adjusting for age, sex and left bundle branch block.

**Conclusions:**

For nonischemic DCM patients with LVEF < 35%, LVRI ≥ 7.5 was associated with lethal VTA events and provided incremental value over conventional CMR parameters.

**Critical relevance statement:**

The left ventricular remodeling index (LVRI) was independently associated with ventricular tachyarrhythmias in dilated cardiomyopathy patients with LVEF < 35%, and warrants future multicenter validation to assess incremental value over established predictors for implantable cardioverter-defibrillator decision-making.

**Key Points:**

- Left ventricular ejection fraction did not exhibit significant prognostic value for end-stage dilated cardiomyopathy patients.
- Cardiac MRI (CMR)-assessed left ventricular remodeling index (LVRI) ≥ 7.5 was an independent predictor of ventricular tachyarrhythmia.
- LVRI provided incremental prognostic value over conventional CMR parameters.

**Graphical Abstract:**

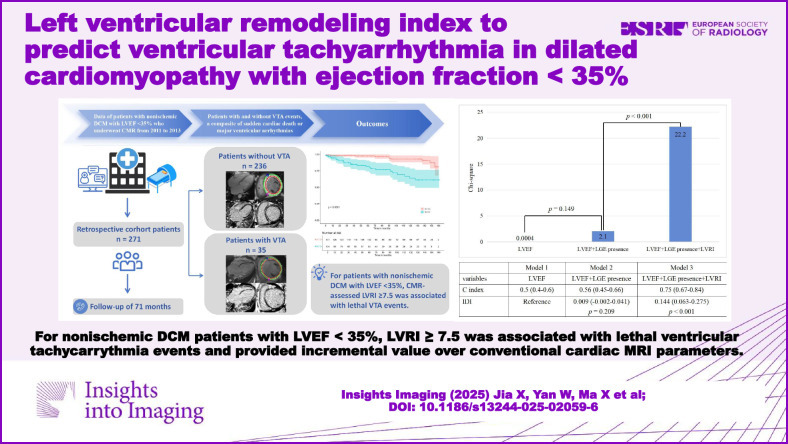

## Introduction

Dilated cardiomyopathy (DCM) is characterized by left ventricular (LV) dilation and systolic dysfunction [1]. Up to 12% of patients experience sudden cardiac death (SCD), accounting for 25–35% of all mortality in DCM [2]. According to the current guidelines [1], an implantable cardioverter-defibrillator (ICD) is well-established to prevent SCD in patients with DCM with LV ejection fraction (LVEF) < 35%. However, studies [3, 4] have demonstrated that LVEF is inadequate for predicting SCD in DCM. Up to 25% of patients receive inappropriate ICD therapy, leading to increased medical costs and patient complications [5–7]. In addition to LVEF, prior studies [8, 9] have shown the prognostic value of late gadolinium enhancement (LGE) in DCM by the use of cardiac magnetic resonance (CMR) [10]. Nevertheless, in the cohort of DCM patients with LVEF < 35%, isolated LVEF and LGE may not exhibit a significant difference between those with and without adverse outcomes [11]. In such circumstances, morphological parameters may provide additional prognostic value for DCM patients with LVEF < 35%. Therefore, it is essential to identify strong risk factors for SCD and who may benefit from ICD implantation in such patients.

The LV remodeling index (LVRI), first proposed by Goh et al [12] in 2017, is deduced from the Law of Laplace and defined as the ratio of the cubic root of the LV end-diastolic volume (LVEDV) to maximal LV wall thickness (LVWT). LV dilation in DCM typically presents as spherical dilation, often accompanied by a significantly thinner ventricular wall. A previous study [13] has shown that LVRI is an independent predictor of all-cause mortality and heart failure (HF)-related events across a wide spectrum of patients with DCM. However, there is no current data regarding the relationship of LVRI with ventricular tachyarrhythmia (VTA) in DCM patients with LVEF < 35%.

Accordingly, the purpose of our study was to explore the predictive value of LVRI, a CMR-derived geometry parameter, for VTA in patients with nonischemic DCM with LVEF < 35%.

## Materials and methods

### Study population

**Fig. 1:**
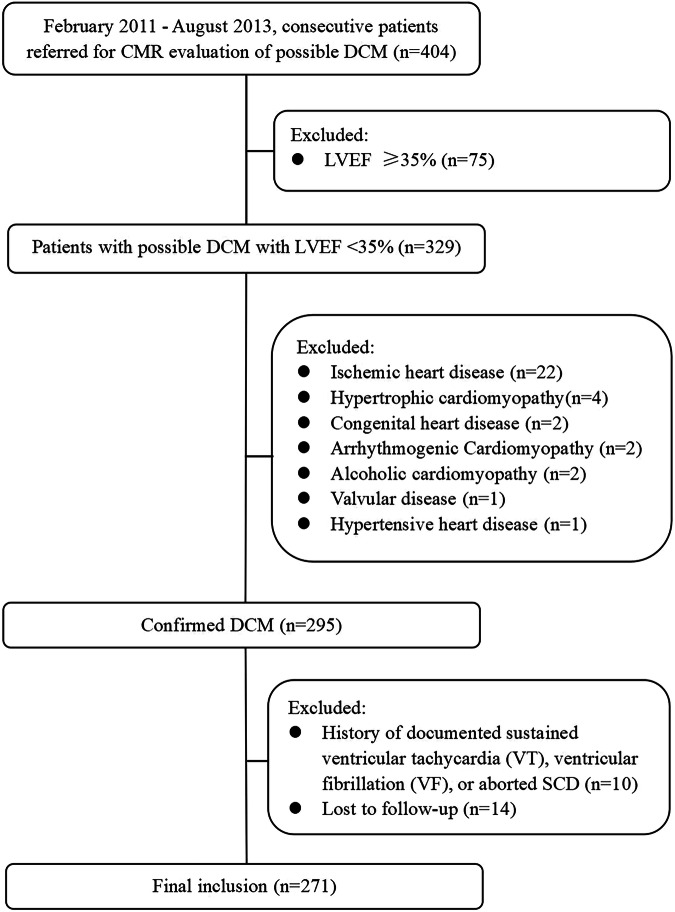
Study cohort. CMR, cardiac magnetic resonance; DCM, dilated cardiomyopathy; LVEF, left ventricular ejection fraction; SCD, sudden cardiac death

This retrospective study conformed to the principles of the Declaration of Helsinki and was approved by the ethics committee of Fuwai Hospital (2021-1590). Written informed consents were obtained from all participants included. In this single-center study, we enrolled consecutive patients with DCM who underwent CMR scans between February 2011 and August 2013 (Fig. 1). The inclusion criterion was the diagnosis of DCM based on a reduced LVEF and LV end-diastolic volume > 2 standard deviations (SDs) from normal according to nomograms corrected by body surface area and age [14, 15]. The exclusion criteria included (1) patients with LVEF ≥ 35%; (2) ischemic heart disease (defined as luminal stenosis > 50% in a major coronary artery identified by angiography, or the presence of ischemia, prior infarction or an ischemic pattern of LGE on CMR imaging), hypertrophic cardiomyopathy, primary valvular disease, congenital heart disease, LV non-compaction, arrhythmogenic cardiomyopathy, alcoholic cardiomyopathy, hypertensive heart disease, cardiac sarcoidosis, and inflammatory myocardial disease; (3) patients with history of documented sustained ventricular tachycardia (VT), ventricular fibrillation (VF), or aborted SCD; and (4) lost to follow-up. Baseline data of demographic characteristics, physical examinations, 12-lead electrocardiogram, medical history, and CMR images were collected at enrollment.

### CMR imaging protocols

All CMR scans were performed with a 1.5-T scanner (MAGNETOM Avanto, Siemens Healthineers) with a phase-arrayed cardiovascular coil and respiratory electrocardiograph gating. Cine images in standard two-, three-, and four-chamber long-axis views and a series of short-axis slices covering the entire ventricle were obtained by a balanced steady-state free precession (b-SSFP) cine sequence with breath-hold. Typical CMR parameters were as follows: slice thickness = 8 mm, slice gap = 2 mm, repetition time (TR) = 3.3 ms, echo time (TE) = 1.7 ms, matrix size = 192 × 224, field of view = 320 × 320 mm, temporal resolution = 46–60 ms, flip angle = 50°. LGE images were acquired 10–15 min after intravenous administration of gadolinium‐DTPA (0.2 mmol/kg, Magnevist, Bayer). Using a phase‐sensitive inversion recovery Turbo Fast Low Angle Shot sequence, LGE images were obtained in the same planes as the end-diastolic cine images. Typical CMR parameters were as follows: slice thickness = 8 mm, slice gap = 2 mm, TR = 8.6 ms, TE = 3.36 ms, matrix size = 256 × 162, field of view = 380 × 320 mm, flip angle = 25°.

### CMR analysis

All CMR scans were analyzed using CVI42 (Circle Cardiovascular Imaging Inc.) by two radiologists (X.J. and W.Y., with 4 and 6 years of CMR imaging experience, respectively) who were blinded to the clinical information. The epicardial and endocardial contours were manually drawn on end-systolic and end-diastolic short-axis cine images. The papillary muscles and trabeculae were excluded from the cavity. Cardiac structural and functional parameters, including left atrial end-diastolic diameter (LAEDD), LV end-diastolic diameter (LVEDD), LVEDV, LVEDV index (LVEDVI), LV end-systolic volume index (LVESVI), LV mass index (LVMI), LVWT, and LVEF, were calculated automatically from the short-axis cine images. LV mass-to-volume ratio (LVMVR) was defined as the ratio of LVM to LVEDV. LVWT represents the maximal wall thickness of 16 myocardial segments [12, 16]. LVRI was subsequently calculated as the ratio of the cubic root of LVEDV to LVWT (LVRI = [∛LVEDV]/LVWT). The presence of LGE was assessed visually by two independent observers mentioned above. LGE presence was regarded as an enhancement signal in 2 phase-encoding directions and both long- and short-axis planes. The LGE patterns were classified as midwall and other LGE. If present, LGE extent was quantified in the short-axis images using the full width at half maximum method and contours were manually adjusted when needed [17].

### Follow-up

The primary endpoint was VTA events, including sustained VT, VF, SCD, and aborted SCD. Sustained VT was defined as an episode lasting ≥ 30 s with ventricular rate ≥ 100 bpm or requiring intervention for termination. VF was defined as a chaotic rhythm with undulations that are irregular in timing and morphology, without discrete QRS complexes on the surface electrocardiograph (ECG). SCD was defined as sudden natural death occurring within 1 h of the onset of cardiac symptoms in witnessed cases, during sleep, or within 24 h of last being seen alive in unwitnessed cases [2]. Aborted SCD was defined as an appropriate ICD shock for ventricular arrhythmia, or the occurrence of nonfatal VF or spontaneous sustained VT leading to hemodynamic compromise and requiring cardioversion [18]. Follow-up data were extracted from hospital records, clinic visits, and telephone interviews by two independent radiologists (X.J. and W.Y., with 4 and 6 years of CMR imaging experience, respectively). The follow-up was continued until July 2024. Time to event was analyzed as the period between the CMR scans and the occurrence of the primary endpoint. Patients who did not experience the primary endpoint were censored at the time of last follow-up.

### Statistical analysis

Continuous data were expressed as mean ± SD or median (interquartile range). Categorical data were presented as numbers and percentages. Normality test was performed using the Kolmogorov–Smirnov method. Parameters of the two groups were compared using the independent-sample t test for normally distributed variables, the Mann–Whitney U test for non-normally distributed variables, and the χ^2^ test or Fisher’s exact test for categorical variables, as appropriate. Correlations between two parameters were analyzed using Pearson’s and Spearman’s coefficients for normally and non-normally distributed variables, respectively. Receiver operating characteristic (ROC) curve was used to calculate the optimal cutoff value of LVRI for the primary endpoint using the Youden index. Prognostic accuracy was assessed by the area under the curve (AUC). Event-free survival curves of two groups of patients, stratified by the cutoff value of LVRI, were estimated by the Kaplan–Meier method and compared by the log-rank test. Considering heart transplantation and HF-related death as competing risks, the competing risk regression was analyzed for VTA events by the Fine and Gray method [19]. Age, sex, and variables with statistical significance (*p* < 0.05) in univariable analyses were enrolled in the multivariable analyses. Additionally, variables with variance inflation factor greater than 5 were excluded from the multivariable analyses to avoid collinearity. Two separate multivariate models were created for the analyses of LVRI as categorical (model 1) and continuous (model 2) variable. Hazard ratios (HRs) with corresponding 95% confidence interval (CI) were calculated. The internal validation was performed using non-parametric bootstrap resampling with 1000 iterations. The incremental values of incorporating LVRI into Cox models were evaluated by calculating the improvement of Chi-square values and C-index. Reclassification of patients by adding LVRI to the baseline model was further evaluated by integrated discrimination improvement (IDI). Interobserver variabilities of LVWT parameter were evaluated by intraclass correlation coefficients (ICC). Two-tailed *p*-value < 0.05 indicated statistical significance. Statistical analyses were performed with SPSS (version 26; IBM) and R (version 4.4.1) statistical software.

## Results

### Demography and baseline characteristics of all participants

**Fig. 2:**
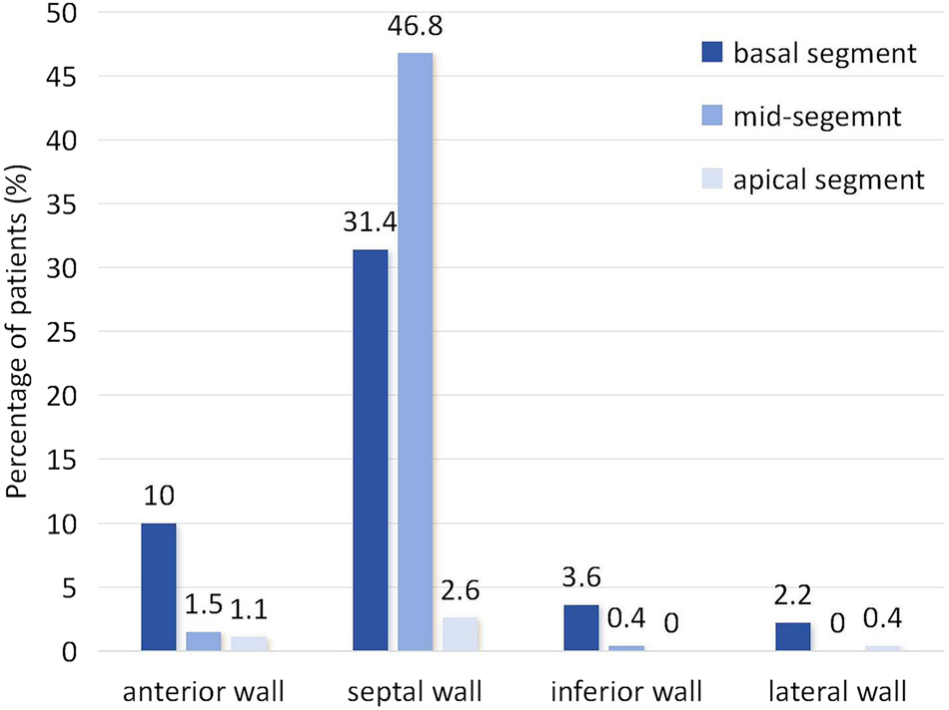
The location distribution of the thickest ventricular wall segments in all patients

**Fig. 3:**
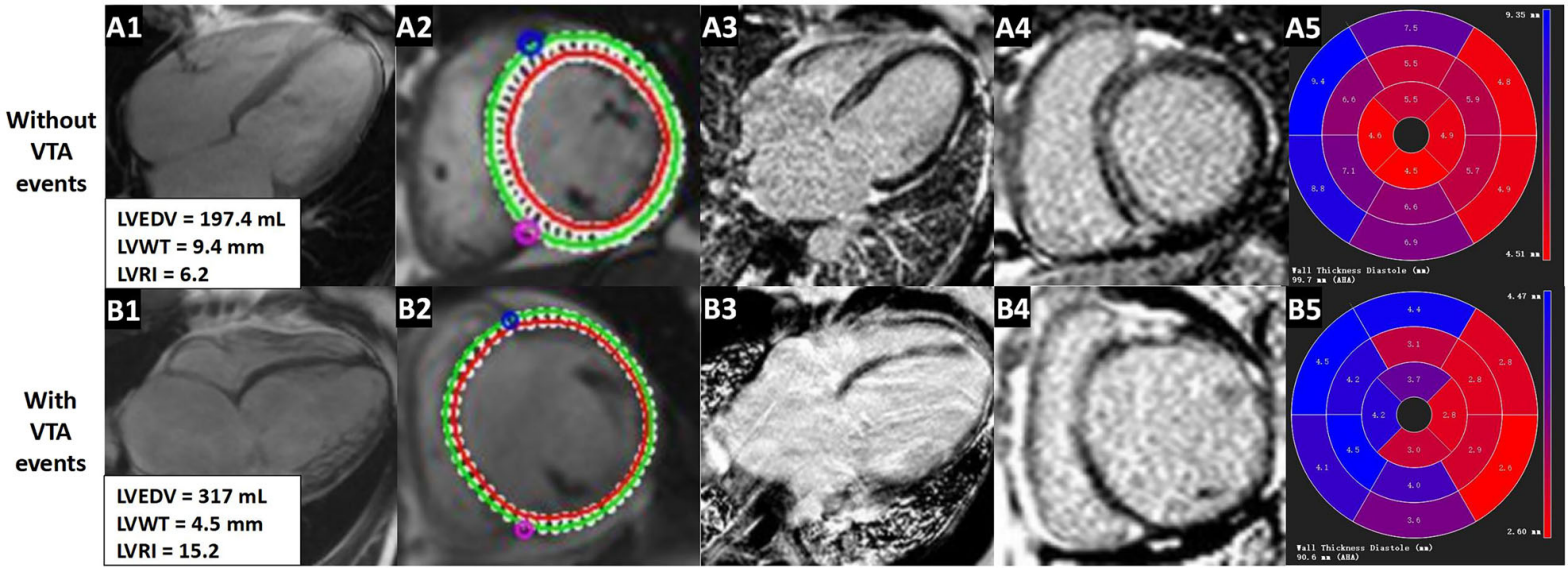
Representative CMR images of two nonischemic DCM patients without (**A**) and with (**B**) VTA events. Cine images (**A1**, **A2**, **B1**, **B2**) and LGE images (**A3**, **A4**, **B3**, **B4**) on four-chamber long-axis views and basal short-axis slices. Images of 16 myocardial segments reflecting the left ventricular wall thickness (**A5**, **B5**). Example cases **A** 47-year-old male with a lower LVRI calculated as 6.2, who had non-VTA events. LGE images reveal typical septal midwall linear enhancement. **B** A 19-year-old female with a higher LVRI calculated as 15.2, who had experienced SCD while having no enhancement on LGE images. LVEDV, left ventricular end-diastolic volume; LVWT, left ventricular wall thickness; LVRI, left ventricular remodeling index; CMR, cardiac magnetic resonance; DCM, dilated cardiomyopathy; VTA, ventricular tachyarrhythmia; LGE, late gadolinium enhancement; SCD, sudden cardiac death

As shown in the flow chart (Fig. 1), a total of 271 patients (age 44.6 ± 14.2 years, 223 (81.9%) men) were enrolled in the final analysis, which included 236 patients without VTA events and 35 patients with VTA events. One hundred and two patients had competing risk events, including HF-related death and heart transplantation. Demographic and baseline characteristics of all patients are summarized in Table 1. There were no statistically significant differences in age, sex, body mass index (BMI), New York Heart Association (NYHA) class, history of hypertension and diabetes between the two groups. Dyspnea (80%) and palpitation (45%) were the most common symptoms. No patients had a history of documented sustained VT, VF, or aborted SCD. LGE was present in 60.5% of (164/271) patients, with the most common pattern of midwall LGE (113/271, 41.7%). The mean LVEF, LVMVR, LGE extent, LVWT, and LVRI of all patients were 22 ± 6.1%, 0.45 ± 0.2, 6.5 ± 8.1%, 8.9 ± 1.8 mm, and 7.7 ± 1.9, respectively. In 80.8% of all patients, the thickest segment of LV myocardial wall was observed in the interventricular septum, including 31.4% in the basal myocardium and 46.8% in the middle myocardium (Fig. 2). Patients with VTA events had significantly higher incidence of left bundle branch block (LBBB) (25.7% vs. 12.2%, *p* = 0.033), lower LVEDVI (145.3 vs. 163.6 mL/m^2^, *p* = 0.028), lower LVMI (60.1 vs. 68.1 g/m^2^, *p* = 0.048), thinner LVWT (7.9 vs. 9 mm, *p* = 0.001), and higher LVRI (8.3 vs. 7.6, *p* = 0.031) when compared to patients without VTA events. In addition, there were no significant differences in LAEDD, LVEDD, LVESVI, LVMVR, LVEF, and the presence, extent and pattern of LGE between patients with and without VTA events (all *p* > 0.05). The representative CMR images of two nonischemic DCM patients without and with VTA events are presented in Fig. 3.

**Table 1:** Clinical characteristics and imaging parameters of all participants

Parameters	All patients (*n* = 271)	Without VTA events (*n* = 236)	With VTA events (*n* = 35)	*p*-value
Age (years)	44.6 ± 14.2	44.3 ± 14.4	46.7 ± 12.7	0.366
Males (%)	223 (81.9)	196 (83)	27 (77.1)	0.393
BMI (kg/m^2^)	24.1 ± 4	24 ± 4.1	24.4 ± 3.3	0.575
SBP (mmHg)	112 ± 17	112 ± 17	110 ± 14	0.504
DBP (mmHg)	72 ± 12	73 ± 12	72 ± 12	0.666
NYHA (%)				0.184
I–II	104 (38.4)	87 (36.7)	17 (48.6)	
III-IV	167 (61.6)	149 (63.3)	18 (51.4)	
Hypertension (%)	86 (31.7)	73 (30.9)	13 (31.7)	0.461
Diabetes (%)	42 (15.4)	36 (15.2)	6 (17.1)	0.773
Smoker (%)	92 (33.8)	82 (43.7)	10 (28.5)	0.472
Alcohol user (%)	74 (27.3)	64 (27.1)	10 (28.5)	0.158
LBBB (%)	38 (14)	29 (12.2)	9 (25.7)	0.033
AF (%)	62 (22.8)	54 (22.8)	8 (22.8)	0.997
Symptoms (%)				
Dyspnea	217 (80)	187 (79.2)	30 (85.7)	0.371
Palpitation	122 (45)	109 (46.1)	13 (37.1)	0.316
Medications (%)				
ACEI/ARB	179 (66)	153 (64.8)	26 (74.2)	0.27
β-blockers	135 (49.8)	117 (49.5)	18 (51.4)	0.838
Diuretics	254 (93.6)	221 (93.6)	33 (94.2)	> 0.99
Anti-coagulation	135 (49.8)	118 (50)	17 (48.5)	0.875
CMR parameters				
LAEDD (mm)	41.1 ± 10.1	41.1 ± 10.2	41.1 ± 9.9	0.988
LVEDD (mm)	72.4 ± 9.3	72.5 ± 9.4	71.5 ± 8.1	0.561
LVEDVI (mL/m^2^)	161.2 ± 57.6	163.6 ± 59.2	145.3 ± 42.4	0.028
LVESVI (mL/m^2^)	127.3 ± 51.4	129.6 ± 53	112.3 ± 36.1	0.063
LVMI (g/m^2^)	67 ± 22.3	68.1 ± 22.7	60.1 ± 18.1	0.048
LVMVR	0.45 ± 0.2	0.45 ± 0.2	0.45 ± 0.2	0.87
LVEF (%)	22 ± 6.1	21.9 ± 10.3	23.3 ± 6	0.197
LGE presence (%)	164 (60.5)	142 (60.2)	22 (62.9)	0.761
LGE pattern				
Midwall	113 (41.7)	99 (41.9)	14 (40)	0.857
Other	51 (18.8)	43 (18.2)	8 (22.9)	0.643
LGE extent	6.5 ± 8.1	6.5 ± 8.3	6.9 ± 6.6	0.747
LVWT	8.9 ± 1.8	9 ± 1.8	7.9 ± 1.6	0.001
LVRI	7.7 ± 1.9	7.6 ± 1.8	8.3 ± 2.1	0.031

*VTA* ventricular tachyarrhythmia, *BMI* body mass index, *SBP* systolic blood pressure, *DBP* diastolic blood pressure, *NYHA* New York Heart Association, *LBBB* left bundle branch block, *AF* atrial fibrillation, *ACEI* angiotensin-converting enzyme inhibitor, *ARB* angiotensin receptor blocker, *CMR* cardiac magnetic resonance, *LAEDD* left atrial end-diastolic diameter, *LVEDD* left ventricular end-diastolic diameter, *LVEDVI* left ventricular end-diastolic volume index, *LVESVI* left ventricular end-systolic volume index, *LVMI* left ventricular mass index, *LVMVR* left ventricular mass-to-volume ratio, *LVEF* left ventricular ejection fraction, *LGE* late gadolinium enhancement, *LVWT* left ventricular wall thickness, *LVRI* left ventricular remodeling index

### Association between CMR parameters and LVRI

**Fig. 4:**
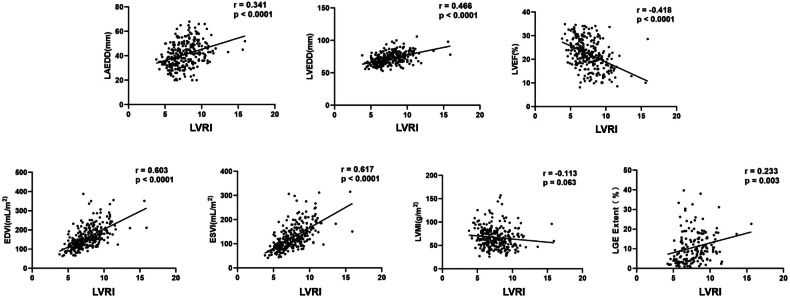
Scatterplots show the correlation between LVRI and LAEDD, LVEDD, LVEF, LVEDVI, LVESVI, LVMI, and the extent of LGE. LVRI, left ventricular remodeling index; LAEDD, left atrial end-diastolic diameter; LVEDD, left ventricular end-diastolic diameter; LVEF, left ventricular ejection fraction; LVEDVI, left ventricular end-diastolic volume index; LVESVI, left ventricular end-systolic volume index; LVMI, left ventricular mass index; LGE, late gadolinium enhancement

The scatterplots indicating the association between CMR parameters and LVRI are shown in Fig. 4. LVRI showed strong correlations with LVEDVI (*r* = 0.603) and LVESVI (*r* = 0.617), and weak correlations with LAEDD (*r* = 0.341), LVEDD (*r* = 0.418), LVEF (*r* = −0.418) (all *p* < 0.0001), and the extent of LGE (*r* = 0.233, *p* = 0.003). However, there was no statistically significant association between LVRI and LVMI (*r* = −0.113, *p* = 0.063), as well as LVRI and the presence of LGE (*r* = 0.065, *p* = 0.287).

### LVRI survival analysis in CMR

During a median follow-up of 71 months (interquartile range: 17–134 months), VTA events occurred in 35 patients, including 12 patients with sustained VT, 1 patient with VF, 4 patients with SCD, and 18 patients with aborted SCD.

**Fig. 5:**
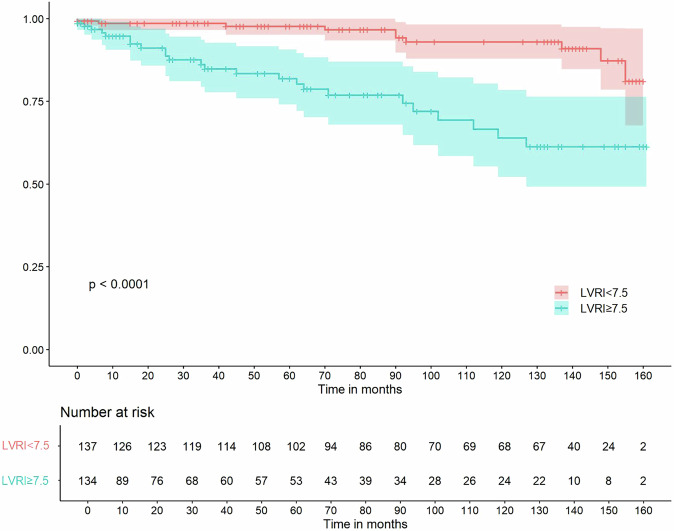
Kaplan–Meier curves for the primary endpoint according to the cutoff value of LVRI (7.5). LVRI, left ventricular remodeling index

The optimal cutoff value of LVRI for the primary endpoint was calculated as 7.5 based on the ROC curve analysis (Supplementary Fig. 1), with 71.4% sensitivity, 53.4% specificity and AUC of 0.61 (95% CI: 0.52–0.71). The Kaplan–Meier curves in Fig. 5 showed that patients with LVRI ≥ 7.5 had significantly worse survival from VTA events (log-rank test *p* < 0.0001). Through 1000 resampling iterations, the bootstrap-derived thresholds showed a median value of 7.7 with an interquartile range of 7.5–7.9, closely clustering around the proposed 7.5 cutoff. Importantly, 75% of the resampled thresholds fell within the clinically reasonable range of 7.5 ± 0.5, suggesting reasonable stability (Supplementary Fig. 2).

The univariable Cox regression analyses demonstrated that LVRI maintained strong associations with VTA events when analyzed both as a dichotomized variable (HR = 4.78, 95% CI: 2.28–10.02, *p* < 0.001) and as a continuous variable (HR = 1.57 per 1-unit increase, 95% CI: 1.30–1.90, *p* < 0.001) in models that did not account for competing risks. The results of univariable and multivariable competing risk regression analyses for VTA events are presented in Table 2. In the univariable competing risk regression analyses, LVRI showed significant associations with VTA events both as a dichotomized variable (≥ 7.5: HR 2.82, 95% CI: 1.37–5.79, *p* = 0.005) and as a continuous variable (per 1-unit increase: HR 1.21, 95% CI: 1.02–1.43, *p* = 0.026), which align well with the univariate Cox regression analyses. In contrast, LVMVR, the presence, extent and pattern of LGE were not associated with VTA events (all *p* > 0.05). In the multivariable competing risk regression model 1, when heart transplantation and HF-related death were counted as competing risks, LVRI ≥ 7.5 (adjusted HR 2.496 [95% CI: 1.213–5.138], *p* = 0.013) was an independent predictor of VTA events. The bootstrap procedure confirmed minimal shrinkage (ΔHR = +1.402%) with a consistent effect size (original HR = 2.496 vs. bootstrapped HR = 2.529, 95% CI: 1.24–6.20) in the primary predictor LVRI. Other clinically relevant variables like LVMI maintained proximate value despite modest shrinkage (7.839%, original HR = 0.983 vs. bootstrapped HR = 0.982, 95% CI: 0.97–1). The bootstrap results (Supplementary Table 1 and Supplementary Fig. 3) support the structural robustness of the original competing risk regression model. However, when LVRI was included as a continuous variable in model 2, the results showed that LVMI was the only independent predictor of VTA events (adjusted HR 0.983 [95% CI: 0.968–0.998], *p* = 0.031), after adjusting for age, sex, LBBB, and LVMI.

**Table 2:** Uni- and multivariable competing risk regression analyses for VTA events with clinical and CMR parameters

Variables	Univariable analysis		Multivariable analysis model 1		Multivariable analysis model 2	
	HR (95% CI)	*p*-value	HR (95% CI)	*p*-value	HR (95% CI)	*p*-value
Age, years	1.01 (0.987–0.99)	0.28	1.176 (0.969–1.427)	0.1	1.01 (0.986–1.033)	0.42
Males	0.662 (0.296–1.48)	0.31	0.852 (0.37–1.962)	0.71	0.874 (0.378–2.018)	0.75
BMI	1.02 (0.952–1.09)	0.63				
SBP	0.991 (0.974–1.01)	0.34				
DBP	0.991 (0.966–1.02)	0.51				
NYHA ≥ III	0.652 (0.337–1.26)	0.2				
Hypertension	1.23 (0.627–2.42)	0.54				
Diabetes	1.18 (0.501–2.77)	0.71				
Smoker	0.728 (0.356–1.49)	0.38				
Alcohol user	1.04 (0.504–2.13)	0.92				
LBBB	2.43 (1.15–5.15)	0.02	2.082 (0.909–4.77)	0.083	2.075 (0.882–4.887)	0.095
AF	1.02 (0.463–2.26)	0.95				
Dyspnea	1.44 (0.552–3.73)	0.46				
Palpitation	0.704 (0.349–1.42)	0.33				
Medications						
ACEI/ARB	1.44 (0.674–3.07)	0.35				
β-blockers	1.12 (0.581–2.17)	0.73				
Diuretics	1.1 (0.264–4.61)	0.89				
Anti-coagulation	1.02 (0.532–1.97)	0.94				
CMR parameters						
LAEDD	1 (0.972–1.03)	0.85				
LVEDD	0.989 (0.957–1.02)	0.49				
LVEDVI	0.994 (0.989–1)	0.046				
LVESVI	0.993 (0.987–1)	0.031				
LVMI	0.982 (0.966–1)	0.034	0.983 (0.968–0.999)	0.033	0.983 (0.968–0.998)	0.031
LVMVR	0.766 (0.142–4.13)	0.76				
LVEF	1.04 (0.982–1.09)	0.19				
LGE presence	1.16 (0.597–2.27)	0.66				
LGE pattern						
Midwall	0.926 (0.474–1.81)	0.82				
Other	1.09 (0.561–2.14)	0.79				
LGE extent (%)	1.01 (0.977–1.04)	0.6				
LVWT	0.68 (0.527–0.878)	0.003				
LVRI ≥ 7.5	2.82 (1.37–5.79)	0.005	2.496 (1.213–5.138)	0.013		
LVRI, per 1 increase	1.21 (1.02–1.43)	0.026			1.176 (0.969–1.427)	0.1

*VTA* ventricular tachyarrhythmia, *CMR* cardiac magnetic resonance, *HR* hazard ratio, *CI* confidence interval, *BMI* body mass index, *SBP* systolic blood pressure, *DBP* diastolic blood pressure, *NYHA* New York Heart Association, *LBBB* left bundle branch block, *AF* atrial fibrillation, *ACEI* angiotensin-converting enzyme inhibitor, *ARB* angiotensin receptor blocker, *LAEDD* left atrial end-diastolic diameter, *LVEDD* left ventricular end-diastolic diameter, *LVEDVI* left ventricular end-diastolic volume index, *LVESVI* left ventricular end-systolic volume index, *LVMI* left ventricular mass index, *LVMVR* left ventricular mass-to-volume ratio, *LVEF* left ventricular ejection fraction, *LGE* late gadolinium enhancement, *LVWT* left ventricular wall thickness, *LVRI* left ventricular remodeling index

### Incremental prognostic values of LVRI over conventional CMR parameters

**Fig. 6:**
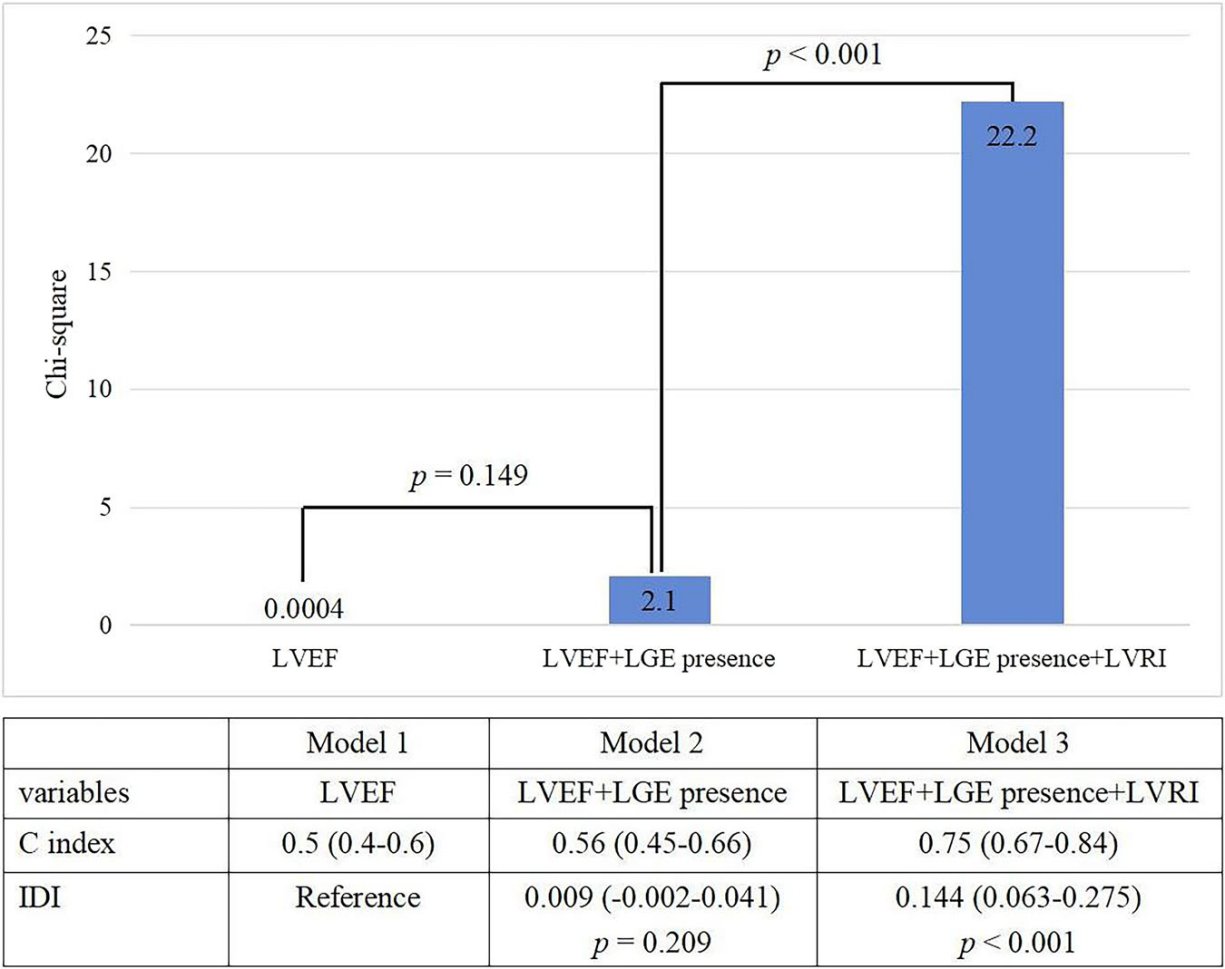
Incremental prognostic value of LVRI over conventional LVEF and LGE presence to VTA endpoints. LVRI, left ventricular remodeling index; LVEF, left ventricular ejection fraction; LGE, late gadolinium enhancement; VTA, ventricular tachyarrhythmia; IDI, integrated discrimination improvement

Different Cox models were utilized to further investigate the prognostic significance of LVRI in predicting VTA among patients with nonischemic DCM with LVEF < 35%. The chi-square values for Model 1 (LVEF), Model 2 (LVEF + LGE presence), and Model 3 (LVEF + LGE presence + LVRI) were 0.0004, 2.1 (*p* = 0.149 vs. Model 1), and 27.1 (*p* < 0.001 vs. Model 2), respectively. The incorporation of LVRI provided significant incremental predictive discrimination (C-index: 0.75 [0.67–0.84]) with clinically meaningful reclassification (IDI = 0.144, *p* < 0.001) (Fig. 6). The analyses of Model 4 (LVEF + LGE extent) and 5 (LVEF + LGE extent + LVRI) were repeated using LGE extent instead of LGE presence. LVRI demonstrated similar incremental predictive value (Chi-square improvement from 3.06 to 20.55, *p* < 0.001; C-index 0.75, 95% CI: 0.67–0.83; IDI = 0.14; all *p* < 0.001), as shown in Supplementary Fig. 4.

### Reproducibility analysis

Quantification of LVWT had good reproducibility in DCM patients, as the interobserver ICC (0.981 [95% CI: 0.953–0.993]) was higher than 0.75.

## Discussion

To the best of our knowledge, this is the first study to investigate the predictive value of LVRI, a CMR-derived geometry parameter, for VTA events in patients with nonischemic DCM with LVEF < 35%. The main findings of the study are as follows: (1) LVRI is strongly correlated with LVEDVI and LVESVI, while weakly correlated with LAEDD, LVEDD, LVEF, and the extent of LGE in CMR; (2) LVRI ≥ 7.5 is an independent predictor of VTA events, including sustained VT, VF, SCD, and aborted SCD, in patients with nonischemic DCM with LVEF < 35%; (3) LVRI provides incremental prognostic value over conventional CMR risk factors, including LVEF, the presence and extent of LGE.

Previous studies [20–24] have demonstrated the predictive value of LV geometry, such as LV sphericity index (LVSI), relative wall thickness, wall curvature, LVMVR and so on, for ventricular arrhythmia in patients with various cardiovascular diseases. Nakamori et al [20] and Levine et al [21] found that LVSI was the independent predictor of ventricular arrhythmias requiring appropriate ICD therapy and was able to increase the predictive accuracy for appropriate ICD therapy in patients with reduced LVEF. Bluemke et al [24] found that the endpoints of incident coronary heart disease and stroke were significantly associated with LVMVR in the Multi-Ethnic-Study of Atherosclerosis. However, LVMVR showed no significant association with VTA events in the current study, highlighting the need for an effective predictor in this cohort. The LVRI, an LV geometry parameter, is calculated by the Law of Laplace to reflect myocardial wall stress [12]. Importantly, LVRI can be easily derived by CMR cine images and without additional scan time or the use of gadolinium contrast. In a prognostic study [16] focusing on patients with hypertensive LV hypertrophy, LVRI was shown to predict adverse events independent of clinical variables and other prognostic factors. Similarly, for DCM patients, Xu et al [13] also found that LVRI was an independent predictor of adverse cardiovascular events and provided incremental prognostic value to LVEF and LGE presence. In the current study, the results revealed that LVRI could independently predict VTA events in patients with nonischemic DCM with LVEF < 35%, which may be explained by several mechanisms. First, the elevated wall stress can augment the dispersion of action potential duration and membrane recovery, thereby altering the electrophysiologic properties of the myocardium [20, 25, 26]. Second, the increased wall stress can serve as a potent stimulus to activate stretch-sensitive ion channels [27, 28]. These dual mechanisms, in concert, contribute to the triggering of VTA.

Additionally, Xu et al [13] have also found that LVRI is significantly associated with myocardial fibrosis, as indicated by native T1 and extracellular volume (ECV) fraction. The paradigm of myocardial fibrosis and scar formation is known to be a substrate for reentry circuits, early afterdepolarizations, and the formation of VTA in DCM patients [29, 30]. Furthermore, fibrosis has been proven to enhance the ability of oxidative stress to induce spontaneous VF [23], which could also explain the findings of our study. Previous studies [20, 31] have demonstrated that LGE on CMR imaging is the gold standard for the assessment of regional myocardial fibrosis. The extent, location, and pattern of LGE have been recognized as independent predictors for SCD-related events across a wide spectrum of patients with DCM [32–34]. However, our data showed that the presence, pattern and extent of LGE were not associated with the occurrence of VTA events in patients with LVEF < 35%. One possible explanation for the discrepancy is that most patients enrolled in this study had progressed to the end stage, resulting in evenly distributed LGE in both VTA group and non-VTA group, with positive rates exceeding 60% and mean LGE extent approaching 6.5% in both groups. Therefore, in the cohort of DCM patients with LVEF < 35%, LVRI may provide additional prognostic value and have additional clinical significance in guiding future ICD implantation and improving patient outcomes.

The study has several limitations. First, being a retrospective single-center study with a limited sample size, it leads to the relatively low incidence of primary endpoint. While the bootstrapping method suggested reasonable coefficient stability, our EPV remains below conventional thresholds. Larger-scale, multicenter, and prospective studies are needed to validate the findings of our study more robustly in the future. Second, most of the patients enrolled have progressed to the end stage of the disease, causing a relatively high incidence of HF-related death and heart transplantation, which may influence some statistical analyses of the primary endpoint. Therefore, heart transplantation and HF-related death were considered as competing risks when the survival analysis was performed. Third, T1 mapping and ECV fraction were not available for all participants, given the retrospective nature of the study. Fourth, the predominance of younger, low-comorbidity patients limits generalizability to older or multimorbid populations, which is a critical target for future validation studies. Lastly, not all patients enrolled underwent genetic testing, and the genetic information was lacking in our study. When combined with CMR parameters, genetic data may provide additional prognostic value, which could be explored in the future.

In conclusion, LVRI ≥ 7.5 is an independent predictor of VTA events in patients with nonischemic DCM with LVEF < 35%. LVRI provides incremental prognostic value over conventional CMR risk factors, including LVEF and the presence of LGE. While LVRI demonstrates strong predictive value in this relatively young and low-comorbidity cohort, its performance in elderly and comorbid patients requires validation through prospective, large-scale, and multicenter studies.

## Supplementary information

ELECTRONIC SUPPLEMENTARY MATERIAL

## Data Availability

All data generated or analyzed during this study are available from the corresponding author on reasonable request.

## Abbreviations

AUC: Receiver operating characteristic
BMI: Body mass index
CI: Confidence interval
DCM: Dilated cardiomyopathy
ECV: Extracellular volume
HF: Heart failure
HR: Hazard ratio
ICD: Implantable cardioverter-defibrillator
LAEDD: Left atrial end-diastolic diameter
LBBB: Left bundle branch block
LGE: Late gadolinium enhancement
LV: Left ventricular
LVEDD: Left ventricular end-diastolic diameter
LVEDVI: Left ventricular end-diastolic volume index
LVEF: Left ventricular ejection fraction
LVESVI: Left ventricular end-systolic volume index
LVMI: Left ventricular mass index
LVRI: Left ventricular remodeling index
LVSI: Left ventricular sphericity index
LVWT: Left ventricular wall thickness
NYHA: New York Heart Association
ROC: Receiver operating characteristic
SCD: Sudden cardiac death
SD: Standard deviation
TE: Echo time
TR: Repetition time
VF: Ventricular fibrillation
VT: Ventricular tachycardia
VTA: Ventricular tachyarrhythmia
