# Supplementary material for: Left ventricular remodeling index to predict ventricular tachyarrhythmia in dilated cardiomyopathy with ejection fraction < 35%
Source: Insights Imaging. 2025 Aug 29;16:188. doi: 10.1186/s13244-025-02059-6 (PMC12397014; doi:10.1186/s13244-025-02059-6)
Supplement: Supplementary file 1 — ELECTRONIC SUPPLEMENTARY MATERIAL [file 13244_2025_2059_MOESM1_ESM.pdf]

Left ventricular remodeling index to predict ventricular tachyarrhythmia in dilated cardiomyopathy with ejection fraction <35%

ELECTRONIC SUPPLEMENTARY MATERIAL

Supplementary Table 1 Bootstrap validation of predictor HRs in the competing risk model

Variable	Original HR	Bootstrap HR	Shrinkage	95%CI	
Age	1.01	1.0099	-0.674	0.99-1.04	
Sex	0.852	0.904	-37.094	0.39-2.78	
LBBB	2.082	2.038	-2.96	0.68-4.96	
LVMI	0.983	0.982	7.839	0.97-1	
LVRI	2.496	2.529	1.402	1.24-6.2	

LBBB, left bundle branch block; LVMI, left ventricular mass index; LVRI, left ventricular remodeling index; HR, hazard ratio; CI, confidence interval

Supplementary Figures

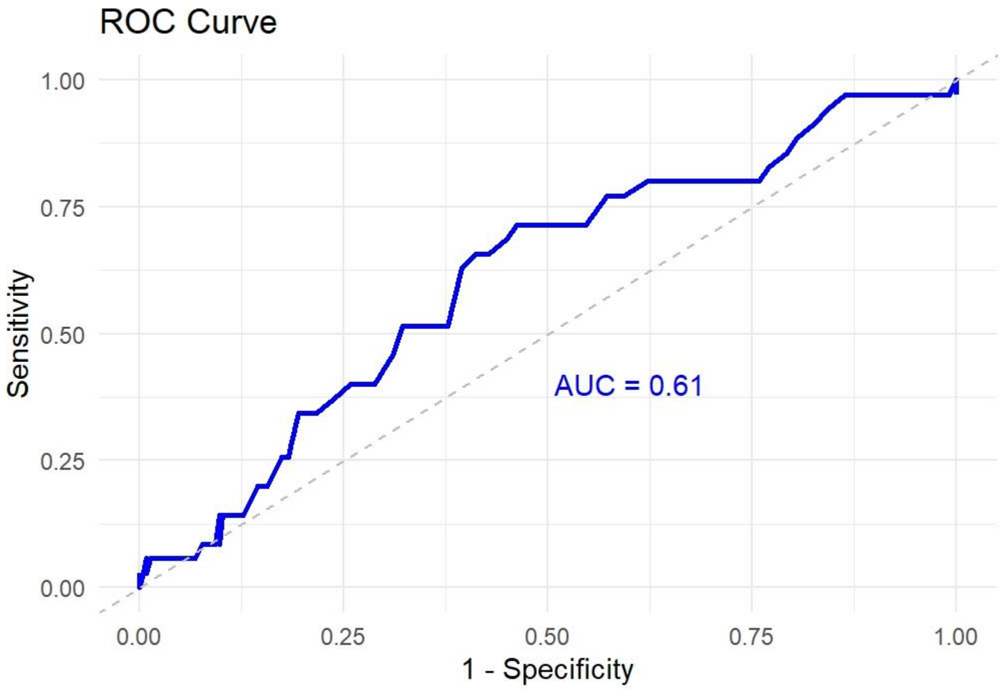

Supplementary Fig.1 ROC curve for association between LVRI and VTA events.

ROC, receiver operator characteristic; LVRI, left ventricular remodeling index; VTA, ventricular tachyarrhythmia

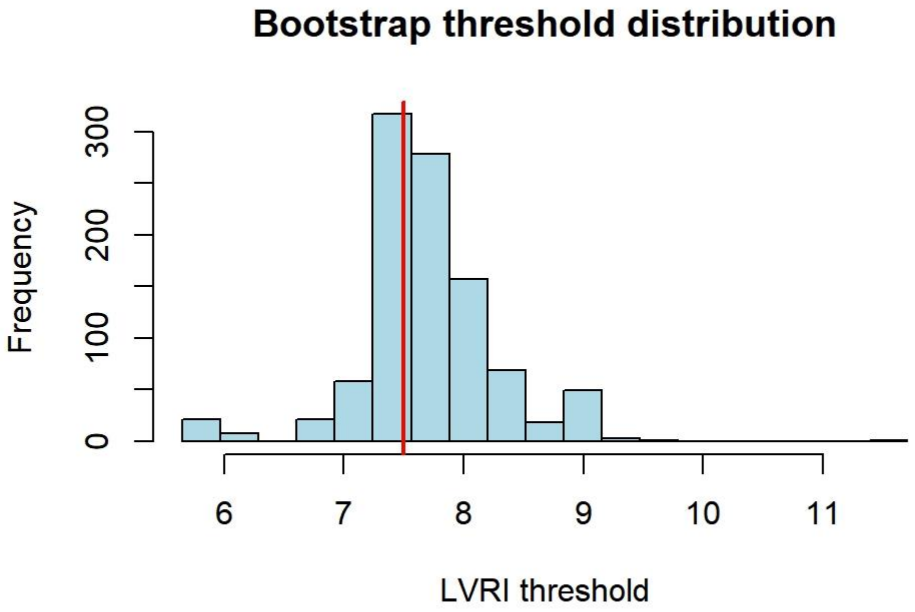

Supplementary Fig.2 Boxplot of LVRI threshold distribution estimated from 1,000 non-parametric bootstrap resamples.

LVRI, left ventricular remodeling index

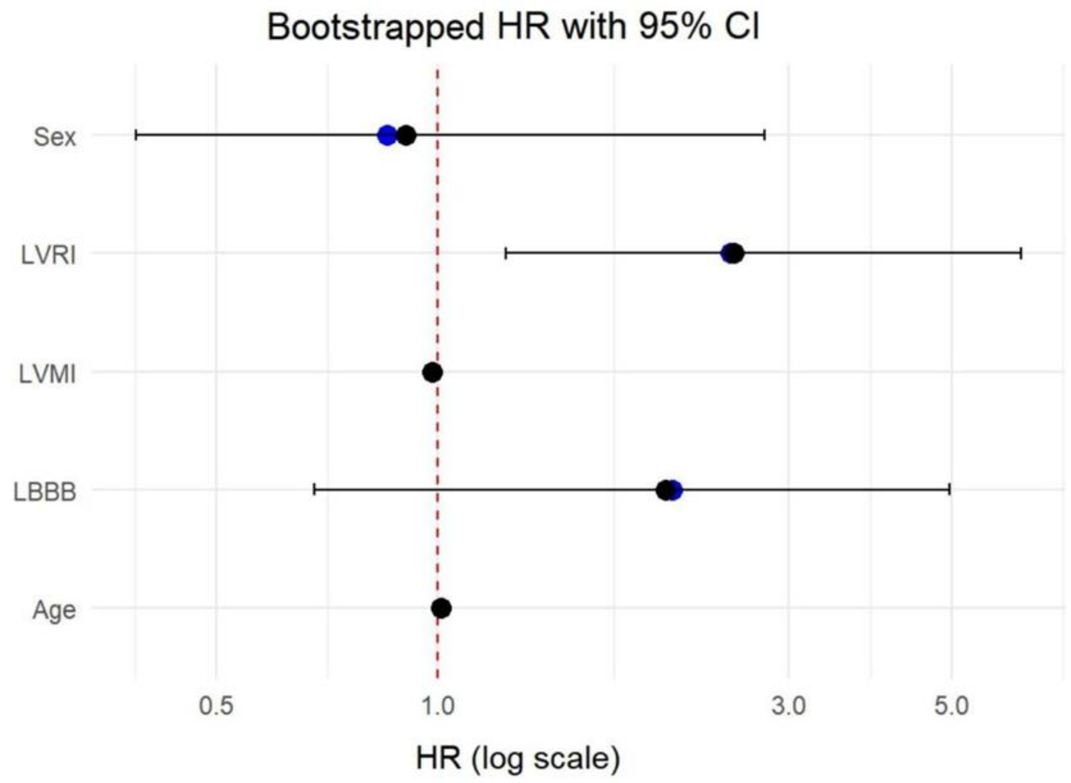

Supplementary Fig. 3 Forest plot of HR and 95% CI derived from 1,000 non-parametric bootstrap resamples.

LVRI, left ventricular remodeling index; LVMI, left ventricular mass index; LBBB, left bundle branch block; HR, hazard ratio; CI, confidence interval

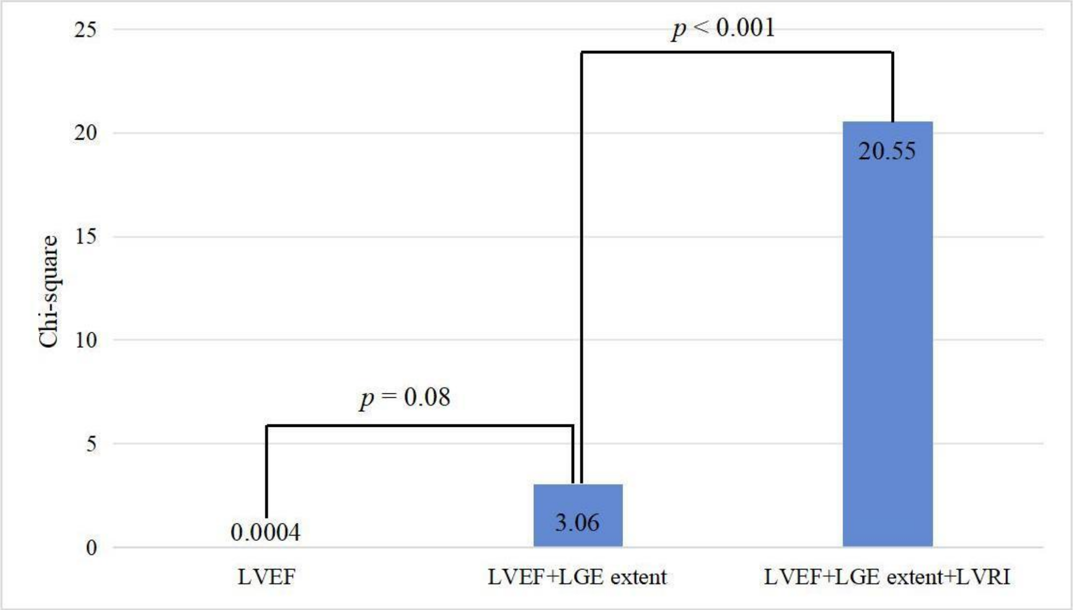

	Model 1	Model 4	Model 5
variables	LVEF	LVEF+LGE extent	LVEF+LGE extent+LVRI
C index	0.5 (0.4-0.6)	0.63 (0.52-0.74)	0.75 (0.67-0.83)
IDI	Reference	0.015 (0-0.053)<br>$p = 0.05$	0.14 (0.063-0.295)<br>$p < 0.001$

Supplementary Fig. 4 Incremental prognostic value of LVRI over conventional LVEF and LGE extent to VTA endpoints.

LVRI, left ventricular remodeling index; LVEF, left ventricular ejection fraction; LGE, late gadolinium enhancement; VTA, ventricular tachyarrhythmia; IDI, integrated discrimination improvement
